# Removal of the Female Urinary Bladder for Malignant Disease1Abstract of a paper read before the Section on Obstetrics, Buffalo Academy of Medicine, May 28, 1901.

**Published:** 1901-07

**Authors:** Matthew D. Mann

**Affiliations:** Buffalo, N. Y.


### REMOVAL OF THE FEMALE URINARY BLADDER FOR MALIGNANT DISEASE.[1]

By MATTHEW D. MANN, A. M., M. D., Buffalo, N. Y.

THE author says the operation for removal of the bladder has not received enough attention in this country, there being very few cases on record. Cancer of the bladder is rare, but is the com-

1. Abstract of a paper read before the Section on Obstetrics, Buffalo Academy of Medicine, May 28, 1901.

monest form of growth found in the bladder. The diagnosis can be made by the symptoms, the use of the cystoscope, palpation, and the examination of the urine. Treatment may be removal through the urethra, the vaginal septum, or by suprapubic cystotomy.

The operations are the removal of the growth and its base; resection of part of the bladder, or cystectomy. Indications for total removal are multiple growths; return after removal; extensive involvement of the base; and extension of cancer of the cervix uteri into the bladder. The ureters need no attention at the time of the operation, as by the removal of a portion of the anterior vaginal wall, they will discharge into the vagina. If possible, the ureteric openings into the bladder should be left intact. This will rarely be possible. He does not believe in uretero-intestinal anastomosis.

The vagina can be used as a receptacle for the urine, as was done by Pawlik. If this be done, there will be little danger of infection traveling to the kidneys, as the newly-made bladder can be kept clean.

The operation is done in the Trendelenburg position. The peritoneum over the bladder being cut, the bladder is enucleated by the fingers, and the base, with the anterior vaginal wall still attached, is removed. The uterus is then removed, and the peritoneum closed over the floor of the pelvis.

Mann reports two cases, both of which recovered from the operation, and has collected from the literature fourteen cases more. He concludes that in certain malignant disease of the bladder, total extirpation is a justifiable operation, offering no serious difficulties to an experienced abdominal surgeon, and giving the patient a chance for a comfortable continued existence.

---

